# Predictors of Transition from Child and Adolescent Bipolar Not Otherwise Specified to Bipolar I Disorder, a Longitudinal 3.9-Year Study

**DOI:** 10.3390/jcm13195656

**Published:** 2024-09-24

**Authors:** María Ribeiro-Fernández, Azucena Díez-Suárez, Kiki D. Chang, Cesar A. Soutullo

**Affiliations:** 1 Department of Psychiatry, Navarra Medical Complex, Navarra Health System (Spanish National Health System), 31008 Pamplona, Navarra, Spain; marietaribeiro@yahoo.com; 2 IdiSNA: Navarra Institute for Health Research, 31008 Pamplona, Navarra, Spain; adiezs@unav.es; 3 Child and Adolescent Psychiatry Unit, Department of Psychiatry & Medical Psychology, University of Navarra Clinic, 31008 Pamplona, Navarra, Spain; 4 Private Practice, Palo Alto, CA 94306, USA; kikichangmd@gmail.com; 5 Louis A. Faillace Department of Psychiatry and Behavioral Sciences, The University of Texas Health Science Center at Houston, Houston, TX 77054, USA

**Keywords:** bipolar, children, adolescent, prevention, diagnosis

## Abstract

**Background:** Children and adolescents with subthreshold manic symptoms not meeting full DSM criteria for bipolar I or II disorder (BP-I or BP-II) are classified as unspecified bipolar disorder (formerly bipolar not otherwise specified: BP-NOS). Factors associated with transition from BP-II or NOS to BP-I may predict the progression of the disorder. Our objective is to analyze factors associated with transition to BP-I in a Spanish sample of youth with BP-NOS or BP-II. **Methods:** We included all youth diagnosed with BP before 18 years of age presenting to our clinic (October 1999–December 2014). We assessed clinical factors that may predict transition to BP I with a logistic regression and a multivariable model for data analysis. **Results:** A total of 72 patients with BP, mean (SD) age 14.5 (10.5–16.0) years, were followed for a median period of 3.9 years. In total, 95.8% of patients retained the BP diagnosis, but they changed type. Baseline BP-I % was 37.5%, and 62.5% at endpoint. BP-NOS decreased from baseline 54.2% to 25% at endpoint. The % of BP-II was 8.3% in both time points, but they were not the same individual patients, as some transitioned from BP-II to BP-I and some BP-NOS changed to BP-II. BP-NOS was stable in 46.1% of patients, but 38.5% transitioned to BP-I over time. Psychotic symptoms during prior depressive episodes (MDD) increased the risk of transition to BP-I by 11-fold. Each individual symptom of mania increased the risk of transition to BP-I by 1.41. **Conclusions:** BP-NOS was stable in 46.1% of patients, but 38.5% transitioned to BP-I over time. Psychotic symptoms during prior MDD episodes increased the risk of transition from BP-NOS to BP-I.

## 1. Introduction

The DSM-5 definition of bipolar disorder (BP) requires at least one episode of mania (or psychosis, or hospitalization during a hypomanic episode) to diagnose BP-I, or at least one depressive episode and one hypomanic episode to diagnose BP-II. Under the bipolar disorders label, DSM-5 also includes cyclothymia, other specified bipolar disorders, and unspecified bipolar and related disorders. When the patient does not meet full criteria for BP-I, II, or cyclothymia (due to an insufficient number of symptoms or insufficient episode duration), they may be considered as BP not otherwise specified (BP-NOS) in DSM-IV terminology or unspecified bipolar disorder in DSM-5 [1,2].

BP in children and adolescents often starts with subsyndromal/subthreshold presentations, often with depressive/mixed features or hypomanic symptoms that do not reach the DSM-5 BP-I threshold for duration or number of symptoms [3,4]. BP not otherwise specified (NOS) lacks the duration (1 week for mania, or 4 days for hypomania) or the number of symptoms (three if the primary mood is euphoria, and four if the mood is irritability) to meet full criteria for BP-I or BP-II. BP-II also requires not meeting the criteria for mania, only hypomania and depressive episodes [1]. However, BP-NOS is associated with equally severe outcomes as BP-I [4] and a 33–59% rate of conversion to BP-I or BP-II, compared with 2% in the general population [2,4,5,6,7,8].

A recent meta-analysis found that children with subthreshold BP had functional outcomes significantly worse than non-BP controls but similar or slightly better than BP-I children [9]. Children with subthreshold BP had significantly more depression and mania symptoms, disruptive behavior-, mood-, and substance use-disorders, and suicidality than non-BP controls. Those with subthreshold BP had similar rates of depression and manic symptoms, and comorbidity (except a lower rate of psychosis and suicidality), as those with full-threshold BP-1. However, there was a similar utilization of mental health services utilization between controls and subthreshold BP, both lower than BP-I [9].

The differential diagnosis, sensitivity, and specificity of these clusters of symptoms are often a challenge to elucidate. The field struggles between increasing the specificity of the diagnosis requiring a full BP-I episode (with the risk of Type 2 error, false negative: delayed diagnosis), or increasing sensitivity and diagnosing BP-NOS or BP-II at earlier stages (with the risk of Type 1 error, false positive: misdiagnosis/overdiagnosis). The purpose of the diagnostic test may inform this choice. A highly sensitive test (with close to 0% false negative rate), despite low specificity, may be preferred for the screening of a condition and a false positive is not a problem. After this test, a more specific test, with low false positive rates, is applied to confirm the diagnosis.

Despite substantial advances in diagnosing youth with BP [10], there is still a need for predictors of disease progression, or worsening of the disorder, from less clearly defined forms, such as BP-NOS or BP-II to the fully syndromal BP-I form. These predictors, and other available tools, such as the University of Pittsburgh Risk Calculator (https://www.cabsresearch.pitt.edu/bpriskcalculator/, accessed on 1 July 2024) [11], may contribute to an earlier diagnosis with higher specificity. Another study in adults developed the Bipolarity Index (BI) that was able to predict conversion from a community and primary care sample of unipolar major depression to BP over 9 years with a specificity of 95.1% and a Negative Predictive Value (NPV) of 97.5, but with low sensitivity (10.2%) and a low Positive Predictive Value (PPV) (5.4). The authors mention the modest PPV, that seems a bit better in patients aged < 30, but they do not mention that in this case, when this BI was negative, it had a 97.5% chance of predicting no conversion to BP [12]. Using the same Netherlands Study of Depression and Anxiety Database, other authors found that anger and aggression reactivity were risk factors for conversion from MDD to BP (hazard ratio 1.4) [13].

Some risk factors in youth for eventual conversion to BP-I have been identified, such as temperamental factors: cyclothymic temperament in youth (mean age 12.7) hospitalized for major depression (MDD) [14], and a high level of impulsivity in BP-NOS [15]; some characteristics in MDD: lower educational level, substance use, younger age at the first MDD episode, and a family history of BP [16]; the onset of depression prior to age 17 [17]; and other factors in BP-NOS: female sex [2], and an early age of onset of BP spectrum symptoms [2,14,18,19,20,21,22]. Some studies identified family history as a risk factor [2,14,16], but others did not [14,22].

More recent studies have identified the rapid onset of symptoms, intense mania, depression, anxiety, mood lability, a family history of (hypo)mania, and the early onset of BP as risk factors to progression from subthreshold forms or BP-NOS to BP-I or BP-II [10]

Thus, there is substantial risk associated with subthreshold BP or BP-NOS, both of conversion to BP-I or BP-II and of negative outcomes (comorbidity and suicidality). In the absence of biological markers, there is still a need for clinical and sociodemographic factors that may predict which patients will eventually develop fully syndromal BP-I, especially in samples outside the U.S.A.

There are very few available studies on the clinical characteristics that may predict conversion to BP-I in youth outside the U.S., especially in European samples. Thus, we wished to study risk factors for patients in our clinic with subthreshold forms of BP to progress to BP-I.

## 2. Methods

The detailed methodology of the study is described elsewhere [22]. Briefly, we included all 72 children/adolescents (age <18 years old) with DSM-IV BP (I, II or NOS) in the Child and Adolescent Psychiatry (CAP) Unit (Outpatient Clinic), University of Navarra Clinic, Pamplona, Spain, from October 1999 to December 2014 (15 years and 3 months). This is a tertiary University Hospital academic outpatient clinic specialized in CAP with a focus on psychopharmacology, research, and teaching. At the time of the study, it was staffed by 1–3 CAP Psychiatrists, a Clinical Psychologist, a Nurse and auxiliary personnel, and trainees (psychiatry residents and child and adolescent psychiatry fellows (1 or 2 at any given time)). We have availability for inpatient admissions. We receive referrals for second opinion from other clinics and hospitals in Spain, Portugal, and other countries (Latin American and some African and central Asian countries) with agreements with the University of Navarra. We also serve as a primary community CAP clinic for all University of Navarra employees, and receive cases from the National Health System as needed. There are no exclusion criteria to be evaluated at our clinic, but the study required a diagnosis of BP. Inclusion criteria were as follows: a DSM-IV or DSM-IV-TR diagnosis of BD, age less than 18 years at the time of diagnosis, and an ability to communicate with the medical team. We excluded patients with neurological disorders or symptoms, other medical conditions, schizophrenia, or intellectual developmental disorder (IQ > 70). This research protocol was evaluated and approved by the Department of Psychiatry and Medical Psychology, the University of Navarra Clinic’s Institutional Review Board, and the University of Navarra College of Medicine PhD Project Committee when the study started in 2004, code: 2004/07/29/79561, and when this analysis was proposed in 2015, code 2015/05/19/76542. We used DSM-IV operational criteria for BP-NOS similar to COBY [2], requiring distinct episodes (elevated, expansive, or irritable mood) that did not meet symptom criteria by 1 symptom or did not meet full duration, but episodes had to have either enough symptoms with not enough duration, or enough duration with not enough symptoms.

We evaluated all patients at the initial clinic visit using the Spanish version of the ‘Kiddie-Schedule for Affective Disorders and Schizophrenia-Present and Lifetime’ (K-SADS-PL) interview template [23] (Ulloa et al., 2006) to assess their current and past symptoms and diagnoses. The same method was used to determine the presence or absence of manic symptoms across the follow-up period. To improve interrater reliability and consistency, we used consensus diagnosis among the 3 authors to clarify the diagnosis at baseline and at all the follow-up visits.

We identified all patients with a diagnosis of BD from 2000 to 2014, either at the initial visit or at any time during the follow-up, and retrospectively evaluated their diagnoses, the persistence or remission of BP, BP subtype, symptoms, and other clinical characteristics present at the last clinic visit. We collected data on demographics, comorbidities, and family psychiatric history, and recorded diagnostic delay (time from symptom onset to diagnosis). Further, we recorded symptoms prior to diagnosis (prodromal), symptoms at the time of diagnosis, and symptoms at the end of follow-up (last available visit). We tracked the subtype of BP at baseline and at the endpoint; the number of psychiatric hospitalizations; pharmacological treatment at follow-up; and treatment response. We then used all these factors to compare patients with BP-NOS who converted to BP-I or II and those who stayed as BP-NOS or recovered (or lost the diagnosis after coming off treatment).

We used a Shapiro–Wilk test to evaluate the normality of the quantitative variables. As most variables had an asymmetric distribution, median and interquartile range (25th to 75th percentiles; IQR) were used as descriptive statistics. The qualitative variables are presented as absolute numbers and percentages. To analyze a priori defined demographic, clinical (phenomenology and comorbidity), and family history factors potentially associated with conversion from BP-NOS or BP-II to BP-I, we performed a logistic regression. Data were captured from their first visit in our clinic, asking in the initial interview about symptoms prior to the onset of BP diagnosis, and the endpoint of the follow-up was the last clinic visit they had with us. That could be a recent visit with us, or a past visit in a patient who never returned to the Clinic and we could not gather information from. In those cases when a patient seem to have dropped out of treatment, we contacted the parents to assess the level of symptoms they had. In one case, when we contacted the parents, they shared that the adolescent had died of suicide a few months after the treatment was discontinued by the family.

We then performed multivariate analysis to correlate individual manic, depressive, or psychotic symptoms and the age at the time of diagnosis with the outcome transition to BP-I. We used IBM SPSS Statistics V20 (IBM Corp. Released 2011. IBM SPSS Statistics for Windows, Version 20.0. Armonk, NY, USA: IBM Corp) and Stata 12.1 (StataCorp 2011. Stata Statistical Software: Release 12. College Station, TX, USA, StataCorp LP).

## 3. Results

We included 72 patients, with a median age (interquartile range: IQR) at the time of BP diagnosis of 13.6 (9.6–15.7) years. Diagnostic stability was 95.8% after a median (IQR) follow-up time of 3.9 (1.8–5.9) years. At baseline, 37.5% of the sample had BP-I, 8.3% BP-II, and 54.2% BP-NOS. At the endpoint, 62.5% had BP-I, 8.3% BP-II, 25% BP-NOS, and 4.2% of patients no longer met criteria for BP. The % of BP-II was 8.3% both at baseline and at the endpoint, but they were not the same patients. Three (7.7%) converted from BP-II to BP-I and three (7.7%) BP-NOS converted to BP-II, so the number of BP-II remained unchanged, but they were different patients.

**Figure 1:**
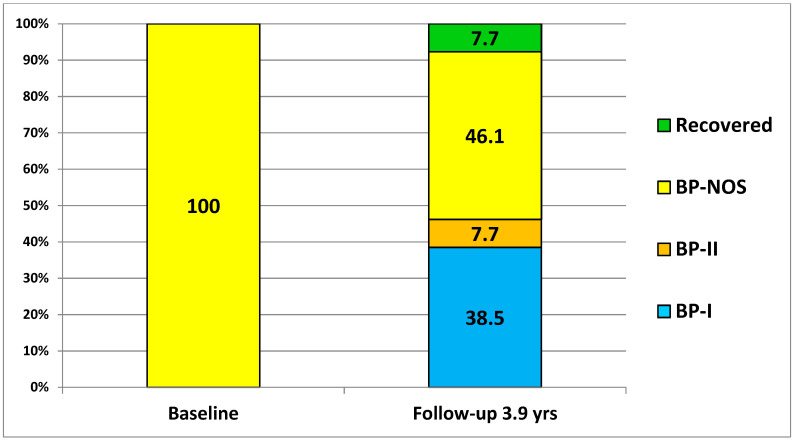
% patients who converted from baseline BP-NOS (N = 39) to BP-I, BP-II, continued as BP-NOS, or recovered over a 3.9-year (47-month) follow-up period (from a total sample of 72 children and adolescents with bipolar disorder).

Of the 39 patients with a baseline diagnosis BP-NOS, 15 (38.5%) converted to BP-I, 3 (7.7%) converted to BP-II, 18 (46.1%) remained BP-NOS, and 3 (7.7%) no longer met criteria for BP and remained off medications for BP at the end of the 3.9-year follow-up period (Figure 1). To clarify, these 3 (7.7%) of the 39 BP-NOS patients are the same 3 (4.2%) of the total 72 patients with BP in the study. Other characteristics have been reported elsewhere [22].

Logistic regression was used to identify factors associated with transition from BP-NOS to BP-I. Variables included were as follows: family history of bipolar or other mood disorder, personal history of substance use disorder, other comorbidity, type of symptoms (grouped by diagnosis: mania, depression, or psychosis; and grouped by domain: cognitive/perception, behavior, and mood), treatment response, and illness characteristics: age at onset and age at first diagnosis and diagnostic or treatment delay.

The logistic regression multivariate model that correlated baseline characteristics with conversion from BP-NOS to BP-I found that the presence of psychotic symptoms (in a prior depressive episode or after the BP diagnosis) multiplied by 11.76 the chances to convert from BP-NOS to BP-I, and that every individual symptom of mania multiplied the risk by 1.41. Age at onset, diagnostic delay, treatment response or delay, and the presence of depressive symptoms had no influence in the conversion from BP-NOS to BP-I (Table 1).

## 4. Conclusions

We found that the vast majority of our patients with subthreshold forms of BP maintained their diagnoses or progressed to BP-I over the course of almost 4 years. Most of the increase in % of BP-I after 3.9 years of follow-up came from conversion from baseline BP-NOS, not from BP-II. BP-I prevalence changed from 37.7% at baseline to 62.5% at follow-up, and BP-NOS decreased from 54.2% at baseline to 25% at follow-up. Interestingly, the % of BP-II was low (8.3%), similar to other samples such as COBY (6.8%) [5,11,24], and patients did not progress from BP-NOS to BP-II and then BP-I, but directly from BP-NOS to BP-I. This phenomenon needs further research, but may be related to the nature of BP-NOS itself.

Psychotic symptoms during any time prior or after the diagnosis multiplied the risk (OR) of transitioning from BP-NOS or BP-II to BP-I by 11.8. Of note, these symptoms were present during major depressive episodes either prior to the diagnosis of BP or after the diagnosis of BP. The presence of each manic symptom increased the risk of conversion to BP-I by an OR of 1.41. These results are consistent with previous studies that found that 25% [23], 38.3% [2], or 52.5% [14] of youth with BP-NOS converted to BP-I or BP-II over 1.8, 4, or 4.5 years, respectively. However, we did not find female sex or an earlier age of onset to be predictive of BP progression as was previously reported [14,24]. We also did not find family history (the presence or absence of BP) to be a risk factor for conversion to BP-I or BP-II, but 12% of our patients were adopted and so family history was unknown [22].

Our main findings indicated that the presence of each individual symptom of mania multiplied by 1.41 the risk of conversion from BP-NOS to BP-I, but we could not identify a specific set of symptoms that were predictive of this conversion. This has important implications since part of the diagnosis effort sometimes consists in defining the duration of episodes, and our data indicates that clinicians should focus more on the number of manic symptoms that are present.

Conversely, the presence of psychotic symptoms (in a prior depressive episode or after the BP diagnosis) multiplied by 11.76 the chances to convert from BP-NOS to BP-I, so this is a strong indicator of a potential risk of conversion. The presence of psychotic symptoms in a prior depressive episode (MDD) may indicate a more sever disorder at baseline, or more severe brain pathology. Youth with a prior episode of MDD with psychotic symptoms, who later develop BP-NOS, are at elevated risk of converting to BP-I.

Patients with BP-II, however, tended to not change their diagnosis. This finding is consistent with studies that found lower rates of conversion from BP-II to BP-I than the rates of conversion from BP-NOS to BP-I, especially in adults (5–7.5%) [18,19,20], but also in adolescents (17.4% [14] and 25% [2]).

The main limitations of the current study are the small sample size, the relatively short follow-up period of 3.9 years, with a high variability of the median (IQR) follow-up time of 3.9 (1.8–5.9) years (25% of the sample had a follow-up of almost 6 years and 75% of the sample at least 1.8 years), and the absence of a time point during adulthood to confirm the persistence of diagnosis. Another limitation is that some data from the study were collected retrospectively, and we did not use the Young Mania Rating scale to track symptom severity, but the K-SADS to assess their presence or absence.

Despite these limitations, this study shows, on an European sample, that bipolar disorder in children and adolescents had a substantial diagnostic stability (95.8%) and was associated with significant poor outcomes, and even the subthreshold forms of the spectrum were associated with a significant risk of progression to more severe fully syndromal forms. Psychotic symptoms and a higher number of manic symptoms were associated with an increased risk of progression to BP-I. The progression from BP-NOS to BP-I did not seem to be associated with the length of the follow-up, nor with diagnostic or treatment delay. These results add to the body of literature on child and adolescent BP-I [10] in its earliest or prodromal stages and reinforce the importance of the early recognition, secondary prevention, and treatment of youth subsyndromal bipolar disorder in a sample outside the U.S. As stated in the literature [10], most of the discussion and efforts over the past 20 years have been on improving specificity on the diagnosis, “defining really well” the criteria so we do not make a false positive diagnosis. However, preventive and early recognition strategies should be more focused on reducing false negatives, including possible early forms of the disorder, that may later improve or worsen. This way, we will not miss opportunities for interventions, much like the strategies in cardiology and oncology. Our data support that even in BP-NOS, not meeting full criteria, at least 38% converted to BP-I, so this is an important diagnosis to make as early as possible.

## Figures and Tables

**Table 1:** Logistic regression multivariate model that correlates symptoms and age at the time of diagnosis with transition from BP NOS to BP-I.

Variables	OR	CI 95%	Sig (LR Test)
Age at BP Diagnosis	0.95	0.74–1.2	0.66
Each Depressive Symptom	1.02	0.7–1.48	0.91
Each Manic Symptom	1.4	0.98–2.01	0.045 *
Psychotic Symptoms	11.76	1.64–84.28	0.006 *

OR: Odds Ratio. CI: Confidence Interval. Sig: Significance. LR: Logistic Regression. *: significant.

## Data Availability

Data are unavailable due to privacy restrictions.
